# Health-related quality of life and distress in cancer patients: results from a large randomised study

**DOI:** 10.1038/sj.bjc.6604789

**Published:** 2008-11-18

**Authors:** B Johansson, Y Brandberg, M Hellbom, C Persson, L-M Petersson, G Berglund, B Glimelius

**Affiliations:** 1 Department of Oncology, Radiology and Clinical Immunology, Uppsala University, Uppsala, Sweden; 2 Department of Oncology-Pathology, Karolinska Institutet, Stockholm, Sweden; 3 Department of Oncology, Lund University Hospital, Lund, Sweden; 4 Department of Clinical Neuroscience, Section for Personal Injury Prevention, Karolinska Institutet, Stockholm, Sweden; 5 Department of Public Health and Caring Sciences, Uppsala University, Uppsala, Sweden

**Keywords:** health-related quality of life, distress, psychosocial support, group rehabilitation

## Abstract

To compare the effectiveness of individual support, group rehabilitation and a combination of the two in improving health-related quality of life (HRQOL) and psychological well-being in cancer patients during 24 months after diagnosis, as compared with standard care (SC). Furthermore, to compare the study sample and a random sample of the Swedish population with regard to HRQOL. A total of 481 consecutive patients, newly diagnosed with cancer, were randomly assigned to one of the four alternatives. Data on HRQOL and psychological well-being were collected at baseline and after 3, 6, 12 and 24 months. The interventions did not improve HRQOL or psychological well-being, as compared with SC. At 3 months, the study sample reported an HRQOL comparable with the normal population. Many cancer patients are able to manage their cancer-related concerns with the support available from SC. However, it is reasonable to assume that the findings suffer from a lack of data from especially vulnerable patients and a possible Hawthorne effect. It cannot be concluded that cancer patients have no need for additional psychosocial interventions. Future projects should include screening and target interventions for those at risk for significant and prolonged psychological distress.

Cancer and its treatment influence health-related quality of life (HRQOL) in several domains, and the assessment of HRQOL is crucial to efforts to improve clinical outcomes (Arndt *et al*, 2004; Stanton *et al*, 2007). Many cancer patients experience psychological distress (Nordin and Glimelius, 1997; Burgess *et al*, 2005; Korfage *et al*, 2006; Strong *et al*, 2007), and a relatively high incidence of post-traumatic stress disorder symptoms has been observed (Kangas *et al*, 2005). A large number of studies have evaluated the effects of psychosocial interventions on psychological distress and HRQOL (van't Spijker *et al*, 1997; Newell *et al*, 2002). However, it is still unclear whether individual or group support is more effective in relieving problems of anxiety and depression (Sheard and Maguire, 1999; Osborn *et al*, 2006). Different types of interventions have been evaluated in different diagnostic groups and at different times in the illness trajectory (Arving *et al*, 2007; May *et al*, 2008), which makes it difficult to assess the clinical value of the trial results. In addition, a majority of the studies include small samples, which jeopardises the possibilities of detecting small but clinically relevant differences between groups.

Techniques from cognitive-behavioural therapy may be effective in relieving psychological distress and treatment side effects in cancer patients (Uitterhoeve *et al*, 2004; Osborn *et al*, 2006). Methods such as distractions, relaxation and cognitive restructuring have been used to reduce, for example anxiety, depression, nausea/vomiting and pain.

Involuntary weight loss is another well-recognised problem affecting HRQOL in cancer patients (Persson *et al*, 2002). This problem may be decreased through early nutritional assessment and subsequent interventions for risk patients (Ravasco *et al*, 2005). Another important aspect of cancer care is the co-ordination between different levels of the care system, to improve continuity and reduce the patients' need for emergency and hospital care (Burge *et al*, 2003).

The Support-Care-Rehabilitation (SCR) project was carried out between 1993 and 1997. The overall aim was to investigate whether individual support begun at diagnosis or group support during the rehabilitation period, or a combination of both, could help relieve short- and long-term physical and psychosocial problems. Patients were randomised among four groups in a 2 × 2 design: (1) individual support (IS) starting immediately, (2) group rehabilitation (GR), starting approximately 3 months after diagnosis, (3) a combination of IS and GR (ISGR) and (4) standard care (SC). The follow-up period was 24 months for all patients.

The aim of this study was to evaluate the effects of IS, GR and ISGR on HRQOL and psychological well-being in cancer patients at 3, 6, 12 and 24 months after diagnosis, as compared with SC. A further aim was to compare the study sample and a random sample of the Swedish population with regard to HRQOL.

## Materials and methods

### Patients

**Figure 1:**
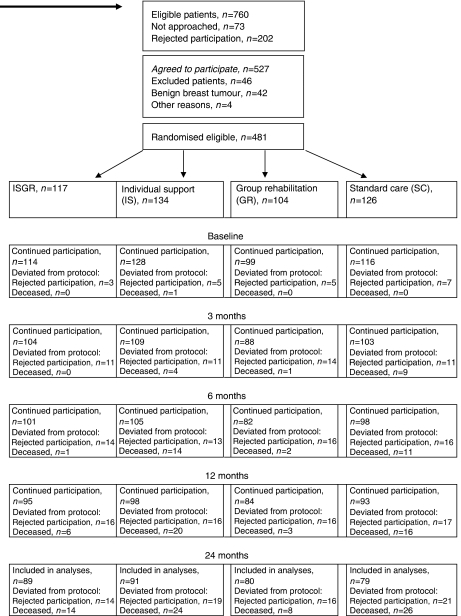
Participant flow.

Consecutive patients with newly diagnosed (<3 months) prostate, gastrointestinal (GI=colorectal or gastric) or breast cancer were included. In addition, women with a mammography finding requiring surgery could also be included. Exclusion criteria were the Karnofsky performance status <40, an earlier cancer diagnosis, inability to communicate in Swedish and participation in an ongoing randomised trial for patients with localised prostate cancer (*n*=59). Of 756 eligible patients, 73 (10%) were not approached due to uncertainty about the diagnosis or administrative failure (Figure 1). A total of 202 patients (26%) rejected participation. The most common reasons for this were ‘no interest’ (*n*=80) and ‘too far to travel’ (*n*=51). Patients who rejected participation were older (mean: 71 years) than those who accepted participation (mean: 62 years, *P*<0.001). This difference was evident in all diagnostic groups. Of the 527 included patients, 42 were excluded due to a benign breast tumour at surgery. Four patients were excluded due to an erroneous cancer diagnosis (*n=*2), senility (*n*=1) or inclusion ⩾3 months from diagnosis (*n*=1). Of all eligible patients, 223 (76%) with breast cancer, 140 (64%) with GI cancer (colorectal, *n*=105 and gastric cancer, *n*=37) and 118 patients (49%) with prostate cancer were included. Thus, of total 756 eligible patients, 481 (64%) agreed to participate, using a procedure approved by the local Research Ethics Committee (Figure 1).

A total of 142 (30%) patients did not complete the entire 24-month follow-up period (Figure 1). Seventy-two (51%) of those died and 70 (49%) rejected further participation. Those who deviated from the protocol were older and included a larger proportion with advanced disease (Advanced disease was GI cancer with distant metastases (M1); prostate cancer with T-stage 4, lymph node and/or distant metastases (N+ and/or M1) and breast cancer with locally advanced cancer (T3–T4) and/or >7 positive axillary nodes or distant metastases (M1).), as compared with those who remained in the project (mean age 67 years compared with 63 years, *P*<0.001; advanced disease *n*=64, 45% compared with *n*=46, 14%, *P*<0.001).

### Randomisation

Randomisation was stratified for diagnosis and stage. Patients were randomised by an independent oncologic centre (computer-generated allocation schedule) to one of the four alternatives. Colorectal and gastric cancer patients who had non-curable disease (M1, short expected survival) were randomised between only IS and SC (no rehabilitation condition). Thus, there was a higher proportion of advanced GI cancer patients in IS and SC, as compared with GR and ISGR (Table 1). With that exception, there were no differences between treatment groups with regard to baseline demographic and medical characteristics.

### Interventions

All interventions have been described in detail elsewhere (Hellbom *et al*, 1998; Johansson *et al*, 1999; Petersson *et al*, 2000; Persson *et al*, 2002).

#### IS

Individual support began as soon as possible after randomisation and consisted of individual psychological support, intensified primary health care and nutritional support. All patients in IS were contacted by a project psychologist (Hellbom *et al*, 1998). Current problems identified jointly by the patient and the psychologist were the focus of the intervention. Techniques used were derived from cognitive behaviour therapy, including relaxation techniques, identification and challenging of negative automatic thoughts and activity scheduling and daily planning. If no problems were identified, the contact was terminated, but the patient was permitted to contact the psychologist when problems arise. Most sessions were conducted face to face at the project agency. However, a number of sessions were conducted by telephone because of long travelling distances or disease- or treatment-related problems. The median number of psychologist contacts was 3 (minimum–maximum: 1–24). The psychologists in the project received regular supervision throughout the study period.

Intensified primary health care meant that each patient was referred to the home care nurse in their neighbourhood (Johansson *et al*, 1999, 2001). The patient's general practitioner (GP) was also informed about the cancer diagnosis and the referral to the nurse. GPs and home care nurses received copies of the medical records each time the patient was discharged from hospital or had visited a specialist outpatient clinic. Education in cancer care was arranged during the course of the trial for nurses and GPs. In addition, GPs and home care nurses with patients randomised to IS were offered supervision by a multi-professional oncology team. Overall, 90% of IS patients reported home care nurse follow-up contacts, as compared with 26% of non-IS patients.

Patients with GI cancer also received nutritional support (Persson *et al*, 2002). A dietician made a dietary assessment including calculation of the dietary intake as soon as possible after diagnosis. After assessment, the dietician gave nutritional advice. When needed, supplements and nutritional enrichment were prescribed. The next assessment was scheduled 2–3 months later. Assessments were conducted by telephone or face to face when the patient had a period of in-patient care or visited the outpatient clinic.

#### GR

Group rehabilitation started approximately 3 months after randomisation and comprised 8 weekly sessions and a booster session after 2 months (Petersson *et al*, 2000). The groups consisted of 3–9 participants. Group rehabilitation implied that the patient visited the project agency. A psychologist, physiotherapist and oncology nurse conducted the meetings. Sessions included cognitive behavioural techniques, light physical training and relaxation. In two of the sessions, a physician presented information about cancer and cancer treatment, and a dietician provided dietary advice. All sessions offered opportunities to disclose and discuss concerns with group leaders and members. Overall, 80% of the patients participated in five or more sessions.

#### IS and GR

Individual support and GR meant that the patients received IS followed by GR. Individual psychological support was (with few *exceptions)* terminated before GR began. A total of 132 (67%) of 196 invited patients participated in GR. A significantly larger proportion of patients randomised to ISGR (42 out of 106) declined GR participation as compared with patients randomised to GR only (22 out of 90) (*χ*^2^=5.10; d.f.=1; *P*<0.05).

#### Standard care

Standard care did not include regular follow-ups by a dietician or medical social worker. However, the patients could be referred to such services if the physician or the nurse judged it necessary or if the patient made a specific request. Psychologists were not available at the surgery or oncology departments. Rehabilitation programmes or support groups were not arranged. Referrals to home care nurses or GPs were rare, as they usually are in routine care (Johansson *et al*, 1999).

### Data collection

#### Background data

Data on patients' age, diagnosis, stage of disease and treatment were collected from the medical records.

#### Points of assessments

The patients completed the baseline assessment before being informed about the randomisation result. Subsequent assessments took place 3, 6, 12 and 24 months after inclusion. The research nurse who informed the patients about the project gave them the baseline questionnaires together with a prepaid envelope. At subsequent assessments, the patients were contacted by one of the investigators by phone. The investigator gave instructions and then mailed the questionnaires, written instructions and a prepaid envelope to the patient. Returned questionnaires were checked for incomplete responses, and in such cases the investigator contacted the patients by phone to complete the questionnaire.

#### Questionnaires

This study includes data from the well-known questionnaires European Organization for Research and Treatment of Cancer-Quality of Life Questionnaire (EORTC QLQ-C30) (version 1) (Aaronson *et al*, 1993; Fayers *et al*, 2001), the Hospital Anxiety and Depression Scale (HADS) (Zigmond and Snaith, 1983) and the Impact of Event Scale (IES) (Horowitz *et al*, 1979). All three were included at all points of assessment.

#### HRQOL in the Swedish population

Normative data on EORTC QLQ-C30 (Michelson *et al*, 2000) were used for the comparison of HRQOL in the study sample and the Swedish population. The EORTC QLQ-C30 was sent to 3919 adults and completed by 3069 (78%), 1619 (53%) women and 1450 (47%) men. The mean age for both genders was 51.2 years.

#### Statistical analyses

The statistical analyses were performed with the SPSS v. 12.0.1. Substitution of missing values was made with the mean of each patient's responses, provided that at least half of the subscale items had been completed (Fayers *et al*, 2001). A one-way ANOVA with repeated measures was used to analyse the effects of IS on HRQOL, anxiety, depression and post-traumatic distress at 3 months. A two-way factorial ANOVA (IS/non-IS, and GR/non-GR) with repeated measures was used to analyse treatment-by-time interactions at 6, 12 and 24 months, using 3-month data as a second baseline. Patients with non-curable GI cancer, who were not randomised to GR, were excluded from the latter analyses. Pairwise comparisons, in case of statistically significant main or interactions effects, were performed with a Bonferroni correction. Additional analyses including age as a covariate in all models were also conducted. However, they did not yield differing results (data not presented). Power calculations were undertaken using a power of 80% with a 0.05 two-sided significance level. A sample size of 75 in each group was required to detect a mean difference of 7.5 (s.d.=23.0) in QLQ-C30 subscales. The corresponding values for the HADS anxiety or depression were a sample size of 57 and a mean difference of 1.5 (s.d.=4.0), and for the IES 72 in each group and mean difference of 3 (s.d.=9.0).

Differences in scores for the EORTC QLQ questionnaires from baseline to the 24-month assessment were interpreted in terms of clinical relevance, according to Osoba *et al* (1998), as small (5–10p), moderate (11–19p) and large (>20p) changes. Improvements in levels of depression, anxiety and distress were interpreted as the number with a lower level of problem according to recommended cutoff scores for the HADS and the IES, respectively. In addition, one-way ANOVA was performed to assess initial differences between groups at baseline and 3 months, and *t*-tests for the assessment of differences between participants and dropouts.

Comparisons with normative HRQOL data were adjusted for gender and age (Hjermstad *et al*, 1998). A one-sample *t*-test was used to compare means between the study sample and the normal population.

## Results

Patients who did not complete all five assessments (*n*=142) had a worse HRQOL at baseline compared with those who did. There were differences with regard to the EORTC QLQ-C30 subscales global health status, physical functioning, role functioning, social functioning, fatigue, nausea and vomiting, pain, dyspnoea, appetite loss, constipation and diarrhoea (mean differences=3–13, *P*<0.001 to <0.05), and with regard to the HAD Depression subscale (means=5.1 and 3.5, *P*<0.001).

Data on the EORTC QLQ-C30 at all points of assessments are presented in Table 2 and data on the HADS and IES in Table 3. There were no baseline differences between IS and SC. Group rehabilitation patients had lower levels of constipation (mean=5.9, s.d.=16.4) at 3 months as compared with non-GR patients (mean=10.7, s.d.=23.5) (F=5.4, d.f.=1, *P*=0.02).

### Interaction effects

Role functioning improved from 3 to 6 and 12 months (mean=80.6, 82.2, 90.6; s.d.=28.0, 29.3, 21.9) and deteriorated slightly at 24 months (mean=85.6, s.d.=28.3) in GR patients, whereas it improved at all points of assessment (mean=85.4–88.1, s.d.=25.4–25.3) in non-GR patients (F=2.7, d.f.=3/954, *P*=0.045). However, there were no statistically significant differences in the pairwise comparisons. Appetite loss decreased from 3 to 6 months (mean=5.1–3.7, s.d.=14.2–11.1), worsened at 12 months (mean=5.8, s.d.=16.0) and decreased again at 24 months (mean=4.1, s.d.=12.7) in GR patients, whereas it improved from 3 to 6 and 12 months (mean=8.1, 4.0, 2.4; s.d.=17.7, 12.6, 10.8) and worsened at 24 months (mean=2.8, s.d.=10.7) in non-GR patients (F=4.1, d.f.=3/963, *P*=0.007). Pairwise comparisons revealed a statistically significant mean difference between GR and non-GR patients at 12 months (mean=5.8 vs 2.4). There were no other treatment-by-time interactions and no interaction effects of IS and GR.

### Main effects of treatments

Individual support patients had a higher level of cognitive functioning (mean=87.4, s.d.=14.2) as compared with non-IS patients (mean=84.0, s.d.=16.4) (F=4.6, d.f.=1/321, *P*=0.046) from 3 to 24 months. There were no other main effects of treatments.

### Effect of time

The levels of problems were generally low with the exception of a slightly deteriorated HRQOL at baseline, as compared with the following points of assessment (Tables 2 and 3). Global quality of life, emotional functioning, cognitive functioning, pain, insomnia, appetite loss, anxiety, depression, intrusion and avoidance improved from baseline to 3 months. Financial difficulties worsened from baseline to 3 months. Global quality of life, physical functioning, role functioning, emotional functioning, social functioning, fatigue, nausea/vomiting, appetite loss, diarrhoea, financial difficulties, and depression improved from 3 to 24 months. Intrusion and avoidance improved from baseline to 3 and 6 months, worsened at 12 months and improved again at 24 months. Pairwise comparisons revealed statistically significant differences between the 12-month assessment and the baseline, 3-, 6- and 24-month assessments.

### Comparison to HRQOL in the normal population

The study participants reported lower HRQOL than the normal population at baseline (Table 4). However, the differences in mean values were small, and at 3 months, the study sample reported an HRQOL equal to that in the normal population. It should also be noted that the study participants reported less pain than the normal population at 3, 6, 12 and 24 months, and higher emotional functioning at 6, 12 and 24 months.

## Discussion

The interventions evaluated in the SCR project did not improve newly diagnosed cancer patients' HRQOL or psychological well-being during a 24-month period after diagnosis, as compared with standard care. There were only a few statistically significant differences between treatment groups despite numerous analyses. The scores of several HRQOL domains rapidly improved with time, and at 3 months after diagnosis, the study sample reported an HRQOL comparable with the normal population.

The sparse interaction and main effects of treatments were small and inconclusive. However, the effects of time revealing an improvement in psychological well-being, pain and global quality of life (Tables 2 and 3) from baseline to 3 months in all treatment groups correspond well with results from earlier studies. These show, using the same measures that unscreened groups of cancer patients go through, a period of relatively rapid improvement with regard to HRQOL, including emotional functioning, after diagnosis and medical treatment (Nordin and Glimelius, 1997, 1998; Arving *et al*, 2007). Thus, the measures used in this study seem to be capable of detecting statistically significant and clinically relevant improvements with regard to these aspects.

Our results suggest that cancer patients generally manage to handle cancer-related concerns and side effects with the support available in standard care. However, several studies have shown that a substantial minority of cancer patients report high levels of distress and deteriorated HRQOL, which are not properly diagnosed and attended to (Burgess *et al*, 2005; Korfage *et al*, 2006). Therefore, there are reasons to explore the validity of our results. The following are noteworthy with regard to the external validity: (1) patients who rejected participation were significantly older than those who accepted participation, and (2) included patients who did not complete all assessments were older and more seriously diseased than those who completed the study. Patients with an advanced disease are likely to suffer more from emotional distress and deteriorated HRQOL and benefit from psychosocial interventions (Uitterhoeve *et al*, 2004). High age in cancer patients is associated with increased morbidity owing to concomitant diseases and impaired physical, social and cognitive functions (Extermann and Hurria, 2007). Hence, our results may suffer from a lack of data from subgroups of especially vulnerable patients, and the validity of our findings might thereby be limited to a group of relatively well-functioning patients. This assumption is supported by the presented data on HRQOL showing mainly improvements over time with only one exception for financial difficulties, which increased from baseline to 3 months, but improved again from 3 to 24 months.

When the SCR project was planned and initiated, the decision was not to screen patients, as this was not recommended at the time. On the contrary, great efforts were made to include as many patients as possible in the studied diagnostic groups, which we also succeeded in doing. However, the importance of screening to identify patients with a need for psychosocial support and to target interventions to such groups has been pointed out in several reports during the past decade (Sheard and Maguire, 1999; Sharpe *et al*, 2004). Thus, a recommendation for future projects aimed at evaluating the effects of psychosocial support should be to include screening and to target interventions only to those at risk for significant distress. On the other hand, there is no current consensus as to how to screen patients in need of an intervention for prolonged emotional distress (Mitchell, 2007).

The project was comprised of extensive interventions with the aim of maximising effects and increasing power. Individual support included individual psychological support, intensified primary health care and nutritional support for some patients, and implied extra contact with at least 2–3 different professionals, irrespective of the patients' need for support. Group rehabilitation comprised a total of nine sessions conducted by a psychologist, physiotherapist and an oncology nurse. Both the individual psychological support and GR implied a visit by the patient to the SCR project agency, which for some patients was more than 100 km away from home. The data collection was also extensive. In addition to the measures included in the presented analysis, patients were asked to complete the Courtauld Emotional Control Scale (Watson and Greer, 1983) (baseline), the Miller Behavioural Style Scale (Miller, 1995) (baseline), the Reaction to the Diagnosis of Cancer Questionnaire (Frank-Stromborg, 1989) (baseline and 3 months), the Mental Adjustment to Cancer Scale (Watson *et al*, 1988) (all points of assessment), the Cancer Rehabilitation Evaluation System Short Form (Schag *et al*, 1991) (3, 6, 12 and 24 months) and another 2–5 project-specific questionnaires during the 24-month follow-up period. In total, the data collection comprised 170–230 questions at each time of assessment. Thus, there is a risk that our efforts to maximise and measure the effects of the interventions meant that it was too strenuous for some groups of patients, with a need of support, to complete participation. This assumption is supported by the fact that older patients tended to reject participation and did not respond to questionnaires to the same extent as younger ones.

A possible Hawthorne effect should also be considered, as all study participants were contacted by phone at each point of assessment, to increase response rate and minimise missing data. The participants were contacted before the questionnaires were sent out, if they did not return them within in 2 weeks, and in cases of missing data on returned questionnaires. Hence, project participation may include numerous contacts with the project group, also for those randomised to standard care.

Comparisons to reference data may also contain some methodological difficulties (Hjermstad *et al*, 1998), that is the EORTC QOL-C30 has been developed for oncological patients and not for use in a normal population. However, it is widely recognised that the use of population-based reference values is relevant and provides an important aid in the interpretation of QOL scales (Michelson *et al*, 2000; Fayers *et al*, 2001).

In conclusion, many cancer patients manage to handle cancer-related concerns and side effects with the support available in standard care. However, our findings seem to suffer from a lack of data from especially vulnerable patients and from a possible Hawthorne effect. Hence, it cannot be concluded that cancer patients have no need of additional psychosocial interventions. It is recommended that future projects include screening and target-feasible interventions for those with the highest risks of significant psychological distress.

## Figures and Tables

**Table 1:** Baseline demographic and medical characteristics of the study sample, *n*=481

	**ISGR, *n*=117**	**IS, *n*=134**	**GR, *n*=104**	**SC, *n*=126**
	***n* (%)**	***n* (%)**	***n* (%)**	***n* (%)**
*Age (years) mean (s.d.)*	64.0 (13.2)	64.4 (12.6)	64.6 (13.5)	62.8 (13.0)
*Gender*				
Female	73 (62)	77 (57)	55 (53)	72 (57)
Male	44 (38)	57 (43)	49 (47)	54 (43)
				
*Breast cancer* a	60 (52)	61 (46)	51 (48)	51 (40)
*T-stage*^b^				
T1^c^	40 (66)	42 (79)	36 (70)	41 (80)
T2	19 (32)	17 (28)	11 (22)	8 (16)
T3	1 (2)	1 (2)	2 (4)	0 (0)
				
*N-stage*^d^				
N0	30 (50)	36 (59)	36 (70)	31 (61)
N1	22 (37)	18 (30)	12 (24)	14 (27)
>7 positive lymph nodes	4 (7)	3 (5)	3 (6)	3 (6)
				
*Treatment in addition to surgery*				
Radiotherapy	53 (88)	52 (85)	43 (84)	46 (90)
Chemotherapy	18 (30)	16 (26)	9 (18)	10 (20)
				
*Colorectal cancer*	22 (19)	29 (22)	22 (21)	31 (25)
*Dukes' stage*				
A	1 (5)	4 (14)	0 (0)	5 (16)
B	12 (55)	10 (34)	13 (59)	11 (35)
C	8 (36)	6 (21)	8 (26)	7 (23)
D	1 (5)	9 (31)	1 (5)	8 (26)
				
*Curative surgical resection*				
Yes	21 (95)	21 (72)	20 (91)	21 (68)
No	1 (5)	6 (21)	2 (9)	10 (32)
Not surgically treated^e^	0 (0)	2 (7)	0 (0)	0 (0)
				
*Treatments in addition to surgery*				
Radiotherapy	6 (27)	8 (28)	10 (45)	7 (23)
Chemotherapy	6 (27)	10 (34)	10 (45)	9 (29)
				
*Gastric cancer*	4 (3)	14 (10)	3 (3)	15 (12)
*T-stage*^b^				
1	0 (0)	2 (14)	0 (0)	2 (13)
2	1 (25)	3 (21)	1 (33)	2 (13)
3	1 (25)	0 (0)	0 (0)	1 (7)
4	0 (0)	1 (7)	0 (0)	1 (7)
				
*N-stage*^d^				
0	0 (0)	3 (21)	1 (33)	2 (13)
1	4 (100)	3 (21)	0 (0)	3 (20)
				
*M-stage*				
0	4 (100)	3 (21)	2 (67)	5 (33)
1	0 (0)	11 (79)	1 (33)	10 (67)
				
*Curative surgical resection*				
Yes	4 (100)	3 (22)	1 (33)	3 (20)
No	0 (0)	9 (64)	2 (67)	9 (60)
Not surgically treated^e^	0 (0)	2 (14)	0 (0)	3 20
*Treatments in addition to surgery*				
Chemotherapy	2 (50)	4 (29)	0 (0)	7 (47)
				
*Prostate cancer*	30 (26)	30 9	29 (28)	29 (23)
*T-stage*^b^				
1	7 (23)	5 (17)	4 (14)	4 (14)
2	12 (40)	11 (37)	16 (55)	10 (34)
3	10 (33)	12 (40)	7 (24)	8 (28)
4	1 (3)	1 (3)	2 (7)	4 (14)
				
*N-stage*^d^				
0	5 (17)	5 (17)	9 (31)	10 (34)
1	2 (7)	5 (17)	3 (10)	3 (10)
				
*M-stage*				
0	23 (77)	23 (77)	24 (83)	22 (76)
1	7 (23)	7 (23)	5 (17)	7 (24)
				
*Primary treatment*				
Prostatectomy	2 (7)	2 (7)	6 (21)	4 (14)
Radiotherapy	3 (10)	6 (20)	3 (10)	7 (24)
Hormonal therapy	7 (23)	10 (33)	5 (17)	9 (31)
No primary treatment	18 (60)	12 (40)	15 (52)	9 (31)
				
Recurrence^f^/progression^g^ during study period	20 (17)	16 (12)	13 (12)	14 (11)
Deceased during the study period	15 (13)	25 (19)	10 (10)	27 (21)

GR=group rehabilitation; IS=individual support; ISGR=IS and GR; SC=standard care.

a No breast cancer patients had distant metastases at inclusion.

b T-stage undefined for 5 breast cancer, 21 gastric cancer and 5 prostate cancer patients.

c Including cancer *in situ*, *n*=17.

d N-stage undefined for 24 breast cancer, 20 gastric cancer and 77 prostate cancer patients.

e Patients with distant metastases.

f Nine patients were affected by a new cancer.

g Progression for prostate cancer only.

**Table 2:** Mean values (s.d.) for EORTC QLQ C-30 subscales for all groups (IS, *n*=242–91; ISGR, *n*=114–89; GR, *n*=99–80; SC, *n*=215–79) at five points of assessment

**Subscales**	**Treatment**	**Baseline**	**3 months**	**Treatment**	**3 months**	**6 months**	**12 months**	**24 months**	**Difference**	**F (*P*-value)^a^**
Global health status (ql)^b^^,^^c^	IS	65 (23)	70 (22)	IS	69 (23)	70 (20)	74 (20)	74 (17)	S	
				ISGR	72 (21)	74 (20)	74 (21)	75 (19)	S	1.0
	SC	63 (23)	70 (20)	GR	71 (19)	72 (19)	73 (19)	75 (19)	M	(0.4)
				SC	69 (21)	71 (21)	70 (23)	74 (22)	M	
										
Physical functioning (pf)^c^	IS	83 (22)	83 (21)	IS	84 (19)	84 (20)	84 (22)	85 (21)		
				ISGR	82 (22)	86 (19)	86 (20)	86 (20)		0.1
	SC	81 (25)	83 (20)	GR	82 (19)	87 (15)	88 (17)	87 (20)		(0.9)
				SC	84 (21)	83 (21)	86 (19)	85 (22)		
										
Role functioning (rf)^c^	IS	80 (31)	80 (30)	IS	82 (30)	83 (28)	87 (25)	86 (28)	S	
				ISGR^d^	78 (29)	82 (30)	87 (24)	84 (29)	S	0.5
	SC	78 (34)	82 (28)	GR^d^	83 (26)	82 (29)	92 (23)	88 (27)	M	(0.7)
				SC	81 (29)	85 (28)	87 (25)	90 (23)	M	
Emotional functioning (ef)^b^^,^^c^	IS	74 (23)	84 (18)	IS	83 (18)	83 (18)	85 (17)	87 (17)	M	
				ISGR	84 (19)	86 (18)	87 (18)	87 (18)	M	0.3
	SC	75 (23)	82 (19)	GR	83 (20)	85 (18)	85 (17)	86 (18)	M	(0.8)
				SC	81 (19)	85 (19)	85 (17)	86 (20)	M	
Cognitive functioning (cf)^b^	IS	84 (20)	86 (18)	IS^e^	87 (19)	86 (19)	86 (15)	88 (16)		
				IS^e^ GR	86 (16)	87 (15)	87 (18)	88 (17)		0.3
	SC	82 (21)	83 (19)	GR	84 (18)	84 (20)	86 (18)	86 (19)		(0.8)
				SC	83 (19)	82 (19)	84 (19)	82 (20)		
Social functioning (sf)^c^	IS	84 (24)	88 (20)	IS	87 (20)	90 (17)	92 (15)	91 (18)	S	
				ISGR	88 (20)	92 (16)	93 (16)	93 (15)	S	0.9
	SC	82 (25)	84 (23)	GR	86 (21)	89 (20)	91 (17)	89 (20)	S	(0.4)
				SC	82 (24)	86 (22)	91 (18)	90 (20)	S	
Fatigue (fa)^c^	IS	33 (27)	30 (24)	IS	29 (24)	27 (24)	21 (22)	21 (22)	M	
				ISGR	30 (24)	24 (24)	22 (22)	21 (21)	S	0.6
	SC	33 (26)	30 (24)	GR	30 (21)	27 (23)	22 (21)	20 (20)	S	(0.6)
				SC	31 (26)	26 (23)	23 (22)	22 (26)	M	
Nausea and vomiting (nv)^c^	IS	6 (13)	7 (15)	IS	6 (13)	3 (10)	2 (7)	3 (9)		
				ISGR	7 (16)	3 (11)	3 (8)	2 (7)	S	0.5
	SC	6 (12)	6 (13)	GR	5 (11)	3 (7)	2 (6)	3 (12)		(0.7)
				SC	6 (14)	4 (9)	3 (8)	3 (8)		
Pain (pa)^b^	IS	24 (27)	17 (23)	IS	18 (25)	17 (23)	17 (20)	15 (20)	S	
				ISGR	15 (20)	14 (19)	17 (22)	13 (21)	M	0.4
	SC	23 (26)	18 (21)	GR	18 (23)	16 (20)	15 (20)	12 (20)	M	(0.8)
				SC	19 (19)	17 (22)	18 (23)	15 (23)	M	
Dyspnoea (dy)	IS	17 (25)	18 (23)	IS	19 (24)	23 (26)	18 (25)	19 (27)		
				ISGR	18 (21)	20 (25)	16 (23)	16 (23)		1.8
	SC	17 (23)	18 (23)	GR	18 (21)	15 (19)	19 (20)	15 (24)		(0.1)
				SC	19 (24)	21 (23)	16 (23)	17 (26)		
Insomnia (sl)^b^	IS	27 (31)	20 (26)	IS	21 (27)	24 (28)	19 (30)	18 (28)	S	
				ISGR	19 (24)	19 (28)	18 (24)	18 (25)	S	0.5
	SC	27 (30)	23 (29)	GR	22 (27)	16 (24)	20 (24)	16 (23)	M	(0.7)
				SC	25 (31)	25 (31)	23 (31)	20 (27)	S	
Appetite loss (ap)^b^^,^^c^	IS	18 (30)	9 (20)	IS	11 (21)	6 (15)	4 (15)	3 (10)	M	
				ISGR^d^	8 (19)	5 (14)	7 (20)	3 (11)	M	0.3
	SC	17 (28)	7 (18)	GR^d^	5 (14)	3 (9)	6 (14)	5 (14)	M	(0.8)
				SC	10 (21)	6 (17)	5 (16)	4 (13)	M	
Constipation (co)	IS	9 (23)	8 (19)	IS	9 (20)	9 (19)	9 (19)	8 (19)		
				ISGR^f^	7 (19)	8 (21)	9 (20)	9 (20)		1.0
	SC	10 (23)	9 (21)	GR^f^	5 (14)	9 (19)	9 (18)	6 (17)		(0.4)
				SC	12 (26)	12 (23)	10 (24)	10 (24)		
Diarrhoea (di)^c^	IS	10 (23)	10 (22)	IS	11 (24)	6 (14)	5 (16)	4 (12)	S	
				ISGR	9 (20)	6 (17)	5 (14)	7 (20)	S	0.7
	SC	11 (22)	10 (20)	GR	9 (18)	7 (17)	6 (14)	6 (14)	S	(0.6)
				SC	11 (22)	8 (19)	6 (16)	8 (17)	S	
Financial difficulties (fi)^c^^,^^g^	IS	5 (16)	8 (21)	IS	9 (23)	6 (20)	5 (20)	5 (19)		
				ISGR	7 (20)	6 (15)	7 (17)	5 (14)		1.0
	SC	6 (17)	12 (26)	GR	9 (21)	7 (17)	5 (13)	5 (19)		(0.4)
				SC	14 (30)	13 (26)	11 (26)	7 (19)	S	

GR=group rehabilitation; IS=individual support; ISGR=IS and GR; SC=standard care.

Statistically significant differences from baseline to 24 months (difference) are given as small (S), moderate (M) or large (L).

a F and *P*-value for IS−non-IS × GR−non-GR × time interaction. d.f.=3/954–963.

b Improved from baseline to 3 months (ql, ef, and pa: *P*<0.001; sl: *P*=0.012; cf: *P*=0.04).

c Improved from 3 to 24 months (rf, sf, fa and nv: *P*<0.001; ql and di: *P*=0.001; ef, ap and fi: *P*=0.003; pf: *P*=0.03).

d Time × GR vs non-GR interaction for rf (*P*=0.045) and ap (*P*=0.007).

e Main effect of IS at 3–24 months (*P*=0.046).

f Statistically significant differences between GR patients and non-GR patients at baseline, *P*<0.05.

g Deterioration from baseline to 3 months (*P*<0.001).

**Table 3:** Mean values (s.d.) for HADS and IES for all groups (IS, *n*=241-91; GR, *n*=99-80; ISGR, *n*=114-89; SC, *n*=212-79) at five points of assessment

	**Treat**	**Baseline**	**3 months**	**Treatment**	**3 months**	**6 months**	**12 months**	**24 months**	**Changed level**	**F (*P*-value)^a^**
*HADS*										
Anxiety^b^	IS	5 (5)	3 (4)	IS	3 (3)	4 (3)	4 (4)	4 (4)	17	
				ISGR	4 (4)	3 (3)	3 (3)	3 (3)	17	1.6
	SC	5 (5)	4 (4)	GR	4 (4)	4 (4)	4 (4)	3 (4)	17	(0.2)
				SC	4 (4)	4 (4)	4 (4)	3 (4)	24	
										
Depression^b^^,^^c^	IS	4 (4)	3 (3)	IS	3 (3)	3 (3)	3 (3)	3 (3)	10	
				ISGR	3 (3)	3 (3)	2 (3)	2 (3)	10	1.1
	SC	4 (4)	3 (3)	GR	3 (3)	3 (3)	3 (3)	2 (3)	6	(0.4)
				SC	4 (4)	3 (4)	3 (3)	2 (3)	10	
										
*IES*										
Avoidance^b^^,^^d^	IS	14 (9)	11 (8)	IS	10 (8)	11 (8)	16 (6)	10 (8)	30	
				ISGR	11 (9)	10 (9)	15 (5)	11 (10)	25	1.3
	SC	15 (9)	12 (9)	GR	12 (9)	10 (8)	15 (6)	10 (9)	29	(0.3)
				SC	11 (8)	10 (9)	15 (5)	9 (8)	24	
										
Intrusion^b^^,^^d^	IS	12 (8)	8 (7)	IS	8 (7)	8 (7)	13 (5)	9 (7)	30	0.3
				ISGR	8 (8)	7 (7)	12 (4)	8 (7)	29	(0.8)
	SC	12 (8)	9 (7)	GR	9 (7)	8 (7)	13 (4)	7 (6)	30	
				SC	8 (7)	7 (7)	12 (4)	8 (6)	29	

GR=group rehabilitation; HADS=Hospital Anxiety and Depression Scale; IES=Impact of Event Scale; IS=individual support; ISGR=IS and GR; SC=standard care.

Changed level is the number of patients that changed level (improved) from baseline to 24 months according to recommended cutoff scores for the HADS and the IES

a F and *P*-value for IS-non IS × GR-non GR × time interaction. d.f.=3/954–963.

b Improved from baseline to 3 months, *P*<0.001.

c Improved from 3 to 6,12, and 24 months, *P*=0.014.

d Improved from 3 to 6 months, worsened at 12 and improved again at 24 months, *P*<0.001.

**Table 4:** Mean values for EORTC QLQ C-30 subscales in the normal population and in the study sample at all points of assessments

**Subscales**	**Reference data**	**Baseline (*n*=457)**		**3 months (*n*=404)**		**6 months (*n*=386)**		**12 months (*n*=370)**	**24 months (*n*=339)**	
Global health status	75	64^a^	M	70^a^	S	72^a^		73^a^	75	
Physical functioning	85	82^a^		83		85		86	86	
Role functioning	85	79^a^		81^a^		83		88	87^b^	
Emotional functioning	83	75^a^	S	83		85^b^		85^b^	86^b^	
Cognitive functioning	87	83^a^		85^a^		85^a^		86	86	
Social functioning	90	83^a^	S	86^a^		89		92^b^	91	
Fatigue	22	33^c^	M	30^c^	S	26^c^		22	21	
Nausea and vomiting	3	6^c^		6^c^		3		3	3	
Pain	21	24^c^		17^d^		16^d^	S	17^d^	14^d^	S
Dyspnoea	18	17		18		20		17	17	
Insomnia	18	27^c^	S	22^c^		21^c^		20	18	
Appetite loss	4	17^c^	M	8^c^	S	5		5	4	
Constipation	6	10^c^		8^c^		9^c^		9^c^	8	
Diarrhoea	5	10^c^	S	10^c^	S	7^c^		6	6	
Financial difficulties	7	5^d^		10^c^		8		7	6	

Statistically significant differences given as small (S), moderate (M) or large (L)

a Higher (better) mean value in the normal population, *P*=0.02–<0.001.

b Lower (worse) mean value in the normal population, *P*=0.03–<0.001.

c Lower (better) mean value in the normal population, *P*=0.04–<0.001.

d Higher (worse) mean value in the normal population, *P*=<0.001.
